# Candidalysin Is Required for Neutrophil Recruitment and Virulence During Systemic *Candida albicans* Infection

**DOI:** 10.1093/infdis/jiz322

**Published:** 2019-08-11

**Authors:** Marc Swidergall, Mina Khalaji, Norma V Solis, David L Moyes, Rebecca A Drummond, Bernhard Hube, Michail S Lionakis, Craig Murdoch, Scott G Filler, Julian R Naglik

**Affiliations:** 1 Division of Infectious Diseases, Harbor-UCLA Medical Center, Torrance, California; 2 Institute for Infection and Immunity, Los Angeles Biomedical Research Institute at Harbor-UCLA Medical Center, Torrance, California; 3 School of Clinical Dentistry, Claremont Crescent, University of Sheffield, United Kingdom; 4 Centre for Host-Microbiome Interactions, Faculty of Dentistry, Oral & Craniofacial Sciences, King’s College London, United Kingdom; 5 Fungal Pathogenesis Section, Laboratory of Clinical Immunology and Microbiology, National Institute of Allergy and Infectious Diseases, National Institutes of Health, Bethesda, Maryland; 6 Department of Microbial Pathogenicity Mechanisms, Leibniz Institute for Natural Product Research and Infection Biology (Hans Knoell Institute), Jena, Germany; 7 Friedrich Schiller University, Jena, Germany; 8 David Geffen School of Medicine at UCLA, Los Angeles, California; 9 Present Affiliation: Department of Metabolic and Vascular Physiology, Baker Heart and Diabetes Institute, Melbourne, Victoria, Australia; 10 Present Affiliation: Institute of Immunology and Immunotherapy, Institute of Microbiology and Infection, University of Birmingham, Edgbaston, Birmingham, United Kingdom

**Keywords:** *Candida albicans*, candidalysin, fungal, systemic, endothelial

## Abstract

**Background:**

Candidalysin is a cytolytic peptide toxin secreted by *Candida albicans* hyphae and has significantly advanced our understanding of fungal pathogenesis. Candidalysin is critical for mucosal *C albicans* infections and is known to activate epithelial cells to induce downstream innate immune responses that are associated with protection or immunopathology during oral or vaginal infections. Furthermore, candidalysin activates the NLRP3 inflammasome and causes cytolysis in mononuclear phagocytes. However, the role of candidalysin in driving systemic infections is unknown.

**Methods:**

In this study, using candidalysin-producing and candidalysin-deficient *C albicans* strains, we show that candidalysin activates mitogen-activated protein kinase (MAPK) signaling and chemokine secretion in endothelial cells in vitro.

**Results:**

Candidalysin induces immune activation and neutrophil recruitment in vivo, and it promotes mortality in zebrafish and murine models of systemic fungal infection.

**Conclusions:**

The data demonstrate a key role for candidalysin in neutrophil recruitment and fungal virulence during disseminated systemic *C albicans* infections.

## Teaser

Candidalysin is a cytolytic peptide toxin secreted by *Candida albicans* hyphae. Here we demonstrate a key role for candidalysin in activating endothelial cells, inducing cytokine secretion and neutrophil recruitment, and in promoting fungal virulence during disseminated systemic *C albicans* infections.

Fungal infections affect one quarter of the world’s population and kill ~1.5 million people each year, surpassing the numbers killed by either malaria, breast cancer, or prostate cancer [1]. One of the most medically important fungi is *Candida albicans*, which causes millions of mucosal infections and contributes to ~400 000 life-threatening systemic infections annually [1–3]. Despite these alarmingly high figures, it is unclear how *C albicans* drives mortality during these disseminated systemic infections.

During hematogenously disseminated candidiasis (HDC), *C albicans* must first cross the endothelial lining of the blood vessels at the site of infection [4]. Once in the bloodstream, *C albicans* is disseminated throughout the host where the fungus must again cross the endothelial lining before colonizing the major organs. As such, the endothelium functions as an integral component of the host defence system to recognize and respond to *C albicans* during systemic infections. One potential mechanism by which *C albicans* crosses the endothelial lining is via induced endocytosis, which is a hypha-dependent process [5]. *Candida albicans* hyphae are endocytosed by numerous human endothelial cells including umbilical, dermal microvascular, and brain microvascular endothelial cells [6–9]. Endothelial cells respond to *C albicans* hyphae via the activation of signaling pathways, including the stress-activated p38 mitogen-activated protein kinase (MAPK) pathway [10], and the secretion of proinflammatory mediators such as CXCL8 (interleukin [IL]-8), tumor necrosis factor (TNF)α, IL-1α, and IL-1β [10, 11]. Transcript profiling experiments indicated that overrepresented genes included those for neutrophil recruitment and signal transduction [10]. Hypha formation is critical for the initiation of these endothelial cell responses because hyphal-defective *C albicans* strains (such as *cph1*∆*/efg1*∆) were unable to induce strong proinflammatory responses [12]. Thus, endothelial cells respond to *C albicans* hyphae through the production of proinflammatory cytokines, which may facilitate clearance through active recruitment of immune cells to the site of infection. Likewise, hyphae are known to trigger pyroptosis and the NLRP3 inflammasome in mononuclear phagocytes [13, 14].

A key pathogenic trait of *C albicans* is its ability to induce cell damage. *Candida albicans*-driven damage of epithelial cells and macrophages is mediated by the peptide toxin, candidalysin [13, 15–18]. *Candida albicans* candidalysin is generated from its parent protein (Ece1p) via sequential enzymatic processing by Kex2/1 and secreted from hyphae [19]. Candidalysin is amphipathic, adopts an α-helical structure, and permeabilizes membranes, resulting in the release of lactate dehydrogenase [15], which is a characteristic of cell damage and membrane destabilization. Candidalysin activates epithelial proinflammatory responses via 2 MAPK signaling pathways (p38/c-Fos and MEK/ERK/MKP1) [20–23], which induce downstream immune responses including neutrophil recruitment and innate Type-17 immunity, which are critical for protection against oral and vaginal candidiasis [15, 16, 24–26]. Furthermore, candidalysin is the hyphal moiety that activates the NLRP3 inflammasome and causes cytolysis in mononuclear phagocytes [13].

Currently, the role of candidalysin in driving systemic *C albicans* infections is unknown. Given that expression of candidalysin is hypha-associated and induces damage, signaling and proinflammatory responses in epithelial cells [18, 27–30], and the NLRP3 inflammasome in macrophages [13], we investigated whether candidalysin drives endothelial cell activation, immune cell recruitment, and mortality during systemic infections. We found that candidalysin activates MAPK signaling and cytokine responses in endothelial cells and promotes neutrophil recruitment and mortality during zebrafish and murine models of systemic *C albicans* infection.

## METHODS

### *Candida albicans* Strains

*Candida albicans* strains used in this study are BWP17+CIp30 (wild-type), *ece1*Δ/Δ (*ECE1*-deficient), *ece1*Δ/Δ+*ECE1* (*ECE1* revertant), and *ece1*Δ/Δ+*ECE1*Δ _184–279_ (candidalysin-deficient) as previously described [15].

### Endothelial Cell Culture and *Candida albicans* Infection

Human adult dermal microvascular endothelial cells ([HDMECs] PromoCell GmbH) were cultured in supplemented MV endothelial cell growth medium (PromoCell GmbH). The immortalized human microvascular endothelial cell line, HMEC-1 (provided by Ades et al [31]), were cultured as described [32]. Cells were incubated at 37°C in 5% CO_2_ and transferred to antibiotic and hydrocortisone-free medium. The HDMECs or HMEC-1 (1 × 10^6^) were cultured for 24 hours and then transferred to serum and antibiotic-free media (SFM) for 18 hours. *Candida albicans* strains were cultured in yeast peptone dextrose (YPD) broth (Oxoid) overnight at 25°C, washed with phosphate-buffered saline (PBS), and resuspended in SFM. For signaling experiments, *C albicans* was added to the endothelial cells in SFM (multiplicity of infection [MOI] of 10) and incubated for 2 hours, otherwise endothelial cells were incubated with an MOI of 0.1 for 24 hours; in both instances, infections were carried out at 37°C in 5% CO_2_. Conditioned medium from endothelial cells was collected, centrifuged, and stored at −20°C.

### Cell Lysate Preparation and Immunoblotting

The HMEC-1 cells were washed with ice-cold PBS, lysed with 100 μL ice-cold Radio-Immunoprecipitation Assay (Sigma-Aldrich) buffer containing phosphatase and protease inhibitors (Pierce) on ice for 15 minutes, and then centrifuged at 10 000 ×*g* at 4°C for 10 minutes. Total protein concentration was analyzed by bicinchoninic acid assay (Pierce). Forty micrograms of total protein of each sample was separated by 10% sodium dodecyl sulfate polyacrylamide gel electrophoresis, proteins were transferred to a nitrocellulose membrane (iBlot; Invitrogen), and immunoblot analysis was performed as previously described [15]. Membranes were incubated with primary antibody: rabbit anti-phospho-MEK1/2 (clone 41G9), anti-phospho-p44/42 (ERK1/2) (clone D13.14.4E), anti-phospho-c-Jun (clone 54B3), anti-c-Fos (clone 9F6) (Cell Signaling Technology) at 4°C overnight, followed by horse radish peroxidase-conjugated secondary antibody and developed by enhanced chemiluminescence. β-actin was used as the housekeeping gene (Sigma).

### Lactate Dehydrogenase Release and Cytokine and Chemokine Analysis

Endothelial cell damage was analyzed by measuring the release of lactate dehydrogenase (LDH) into the culture medium using a CytoTox96 assay kit (Promega). The concentration of CXCL8 within conditioned medium was measured by CXCL8 enzyme-linked immunosorbent assay ([ELISA]; BD Biosciences). For murine studies, kidney homogenates were clarified by centrifugation, and the concentrations of cytokines and chemokines were measured using a multiplex bead array (R&D Systems) as previously described [33]. Myeloperoxidase (MPO) protein levels in kidney homogenates were determined using ELISA, following the manufacturer’s instructions (Hycult Biotech).

### Zebrafish *Candida albicans* Infection Model

Zebrafish maintenance and experimental work was performed in accordance with UK Home Office regulations and the UK Animals (Scientific Procedures) Act 1986. London wild-type zebrafish larvae were maintained as previously described [34]. For infection, methylcellulose-embedded, tricaine-anesthetized dechorionated larvae were injected systemically by direct inoculation into the common cardinal vein at 2 days postfertilization with 500 colony-forming units of *C albicans* in PBS or PBS alone as control (n = 20 zebrafish injected per treatment group). Zebrafish viability was assessed by presence of a heartbeat at 24 hours postinfection.

### Mouse Model of Hematogenously Disseminated Candidiasis 

Animal work was approved by the Institutional Animal Care and Use Committee of the Los Angeles Biomedical Research Institute. Virulence of the *C albicans* strains was tested in the mouse model of HDC using 6- to 7-week-old male and female BALB/c mice (Taconics) as previously described [35]. In some mice, leukopenia was induced by intravenous administration of 5 mg of 5-fluorouracil (Western Medical) on day −1 relative to infection. Enrofloxacin (Victor Medical) was added to the drinking water of these mice to prevent bacterial infection. *Candida albicans* strains were serially passaged 3 times in YPD broth, grown at 30°C at 200 rpm for 16–24 hours at each passage. Yeast cells were washed in PBS, renumerated, and injected intravenously via the lateral tail vein. Immunocompetent and leukopenic mice were infected by injecting 2 × 10^5^ and 5 × 10^4^*C albicans* cells, respectively. Each *C albicans* strain was inoculated into 10 mice (5 male/5 female). Animals were randomly assigned to the different groups. Researchers were not blinded to the experimental groups because the endpoints (survival, fungal burden, cytokine levels) were objective measures of disease severity. For survival experiments, mice were monitored twice daily and moribund mice were humanely euthanized. To determine organ fungal burden, mice were sacrificed after 1 day and 4 days of infection, after which the kidneys, brain, spleen, and liver were harvested, weighed, homogenized, and quantified on YPD. For histology, mouse kidneys were fixed in 10% buffered formalin and embedded in paraffin. Thin sections were cut and stained with Grocott-Gomori methenamine silver stain and imaged by light microscopy.

### Statistics

Data are presented as mean values ± standard deviation of at least 3 independent experiments with each test performed in triplicate unless otherwise stated. Mann-Whitney, log rank, or analysis of variance multiple statistical comparisons were performed with Tukey’s post hoc comparison using GraphPad Prism, version 6.00. Differences between test and control groups were considered significant when *P *< .05.

## RESULTS

### Candidalysin Damages and Induces Immune Activation in Endothelial Cells

**Figure 1.**
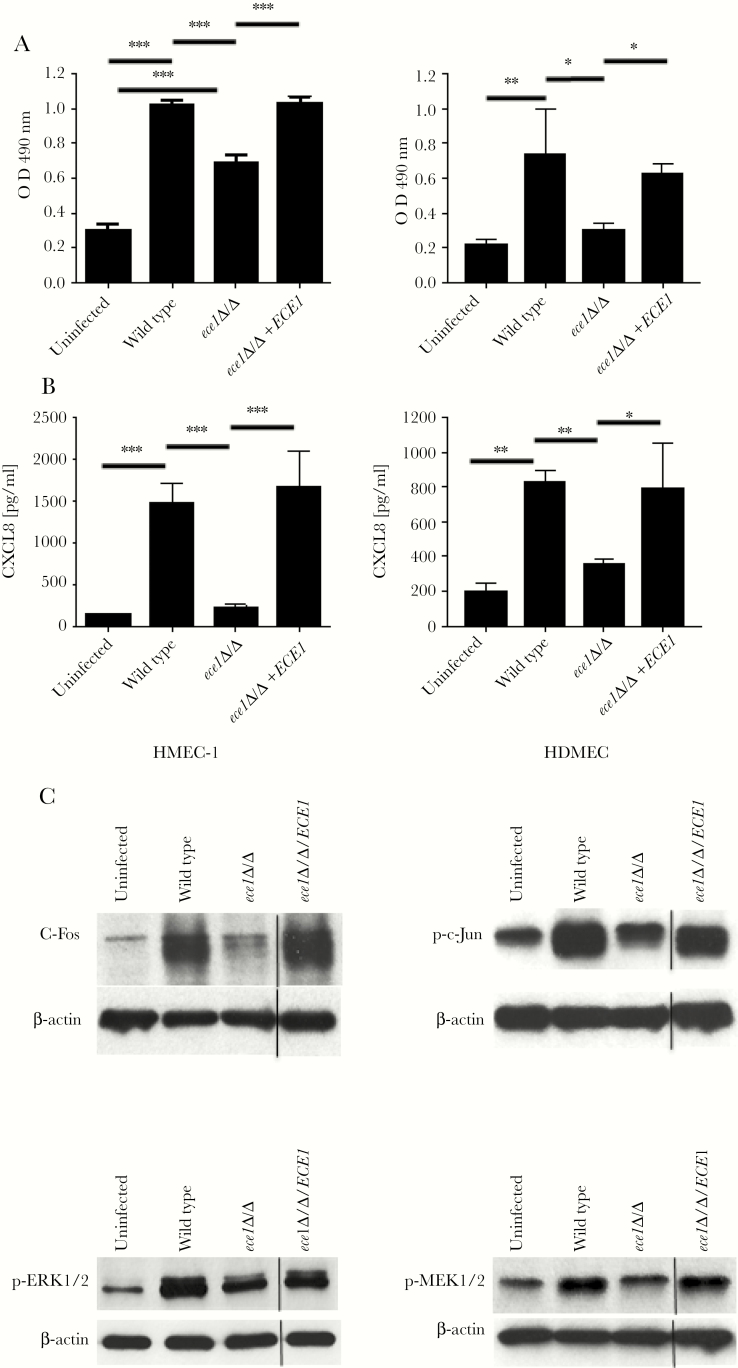
Candidalysin induces cell damage, cytokine release, and intracellular signaling in human endothelial cells. Cell damage (A) and CXCL8 (B) chemokine release quantified by lactate dehydrogenase assay and enzyme-linked immunosorbent assay, respectively, for human microvascular endothelial cell line (HMEC-1) and human adult dermal microvascular endothelial cells (HDMECs) after exposure to wild-type, *ece1Δ/Δ* , or *ece1Δ/Δ+ECE1 Candida albicans* (multiplicity of infection [MOI] of 0.1) for 24 hours; medium alone was used as uninfected control. Data are presented as mean ± standard deviation (n = 3); statistical analysis was achieved using one-way analysis of variance with Tukey’s post hoc multiple comparison test. *, *P *< .05; **, *P *< .01; and ***, *P *< .001. (C) Immunoblot analysis of HMEC-1 intracellular signaling responses to wild-type, *ece1Δ/Δ*, *ece1Δ/Δ+ECE1 C albicans* (MOI of 10) or control for 2 hours. The HMEC-1 cell lysates (40 μg of total protein) were probed with c-Fos, phospho-c-Jun, phospho-ERK1/2, or phospho-MEK1/2, and β-actin was used as a loading control.

One mechanism by which candidalysin may facilitate *C albicans* translocation across the endothelial cell lining to initiate disseminated infection is via the induction of damage. Therefore, we determined whether candidalysin was able to induce LDH release in 2 endothelial cell types. The HDMEC and HMEC-1 cells were incubated with candidalysin-producing (BWP17+CIp30 [wild-type], *ece1*Δ/Δ*+ECE1*) and candidalysin-deficient (*ece1*Δ/Δ; encoding candidalysin) *C albicans* strains, and LDH release was measured after 24 hours. Only candidalysin-producing *C albicans* strains were able to induce LDH release from both endothelial cell types (Figure 1A), indicating that candidalysin induces damage. However, in the HMEC-1 cell line, the *ece1*Δ/Δ mutant was still able to induce a modest amount of damage.

Candidalysin induces cytokine release in epithelial cells and macrophages [13, 15–19, 24, 28, 30]. Therefore, we tested whether candidalysin could also induce cytokine release in HDMEC and HMEC-1 cells. CXCL8 was used as a marker for immune activation because this chemokine is released from endothelial cells in response to *C albicans* [10, 11]. We found that only candidalysin-producing *C albicans* strains were able to induce CXCL8 release (Figure 1B), demonstrating that candidalysin is a critical activator of immune responses in endothelial cells.

Epithelial cells respond to candidalysin activity via the induction of MAPK signaling. Two key proteins that are activated are the AP-1 transcription factor c-Fos (via p38) and the MAPK phosphatase MKP1 (via MEK1/2-ERK1/2) [20–23]. To determine whether candidalysin induces MAPK signaling in endothelial cells, we incubated HMEC-1 cells with *C albicans* strains and tested for c-Fos upregulation and phosphorylation of MEK1/2 and ERK1/2 at 2 hours (previously determined as the optimum time [20]). We additionally tested for the phosphorylation of another AP-1 transcription factor, c-Jun, because this can be activated by JNK (third arm of the MAPK pathway) and can form a heterodimer with c-Fos to activate gene expression. Only candidalysin-producing *C albicans* strains strongly induced c-Fos, phospho-c-Jun, phospho-MEK1/2, and phospho-ERK1/2 in HMEC-1 cells (Figure 1C), indicating that candidalysin is critical for MAPK activation in endothelial cells.

### Candidalysin Is Required for Virulence in a Zebrafish Model of *Candida albicans* Systemic Infection

**Figure 2.**
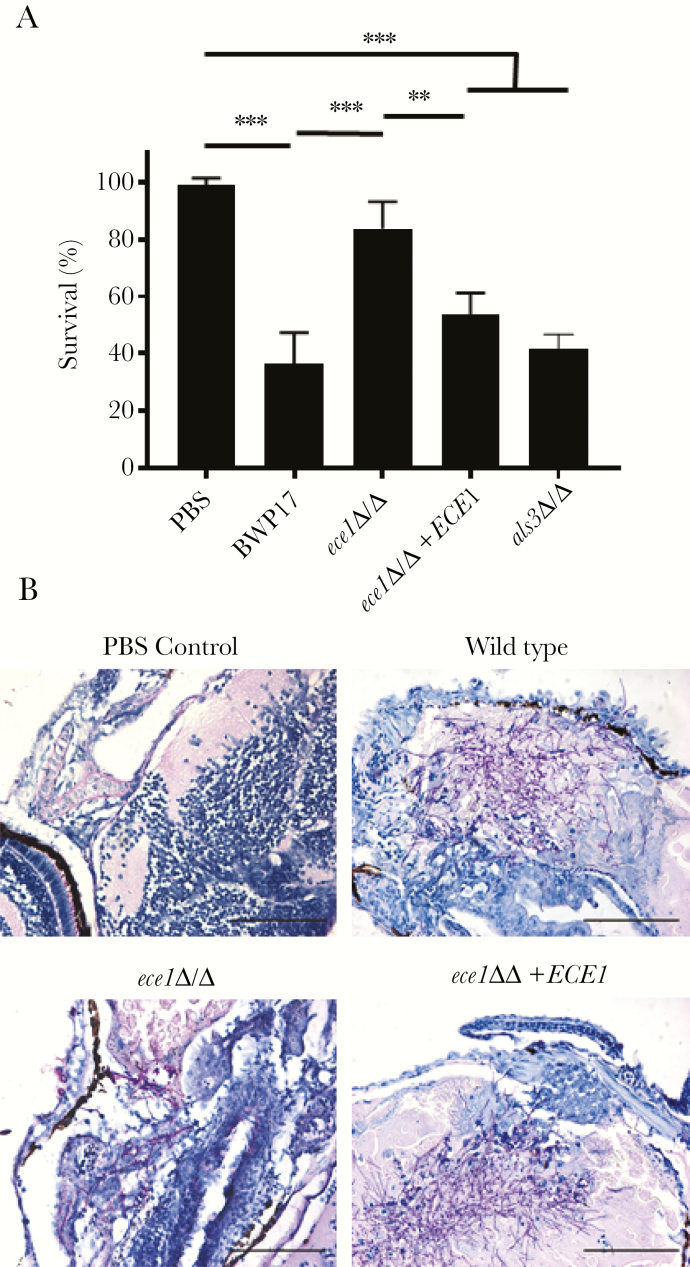
Systemic infection of zebrafish larvae with *ECE1*-deficient *Candida albicans* show improved survival compared with wild-type or *ece1Δ/Δ+ECE1*-infected zebrafish. (A) Zebrafish survival 24 hours after injection of 500 colony-forming unit of wild-type, *ece1Δ/Δ*, *ece1Δ/Δ+ECE1, als3*Δ/Δ or phosphate-buffered saline (PBS) as control directly into the cardinal vein. Data are presented as mean ± standard deviation (n = 4 independent experiments with at least 20 zebrafish per treatment group); statistical analysis was achieved using one-way analysis of variance with Tukey’s post hoc multiple comparison test. **, *P *< .01; and ***, *P *< .001. (B) Sagittal histological sections of periodic acid-Schiff-stained infected zebrafish showing the hyphal form of *C albicans* penetrating zebrafish tissue for wild-type, *ece1Δ/Δ* and *ece1Δ/Δ+ECE1* strains compared with PBS-injected controls. Scale bars = 100 μm.

The ability of candidalysin to damage and induce MAPK-mediated immune responses in endothelial cells may enhance the ability of *C albicans* to translocate across the endothelial lining and promote systemic infection, similar to *C albicans* translocation across epithelial layers [17]. Furthermore, these activities may be associated with protective or detrimental immune responses [16, 24]. To determine whether candidalysin was required for systemic infection, we first utilized a zebrafish model to screen the virulence potential of the *C albicans* strains. Zebrafish larvae were infected with *C albicans* strains in the common cardinal vein, and survival was monitored for 24 hours. Although ~35% of fish survived when infected with candidalysin-producing strains, significantly more (~80%) fish survived when infected with a candidalysin-deficient (*ece1*Δ/Δ) strain (Figure 2A). Histological analysis showed extensive hyphal network formation in fish infected with the candidalysin-producing strains along with widespread tissue destruction and edema. In contrast, in fish infected with the candidalysin-deficient strain, fewer hyphae were present with limited associated tissue damage (Figure 2B). In a control experiment, a *C albicans* strain deficient in the hypha-associated adhesin/invasion *ALS3* (*als3* Δ/Δ) was equally as virulent as the wild-type (Figure 2A). The data indicate that candidalysin promotes infection and a deleterious phenotype in this zebrafish systemic model.

### Candidalysin Is Required for Neutrophil Recruitment and Virulence in a Murine Model of Disseminated *Candida albicans* Infection

**Figure 3.**
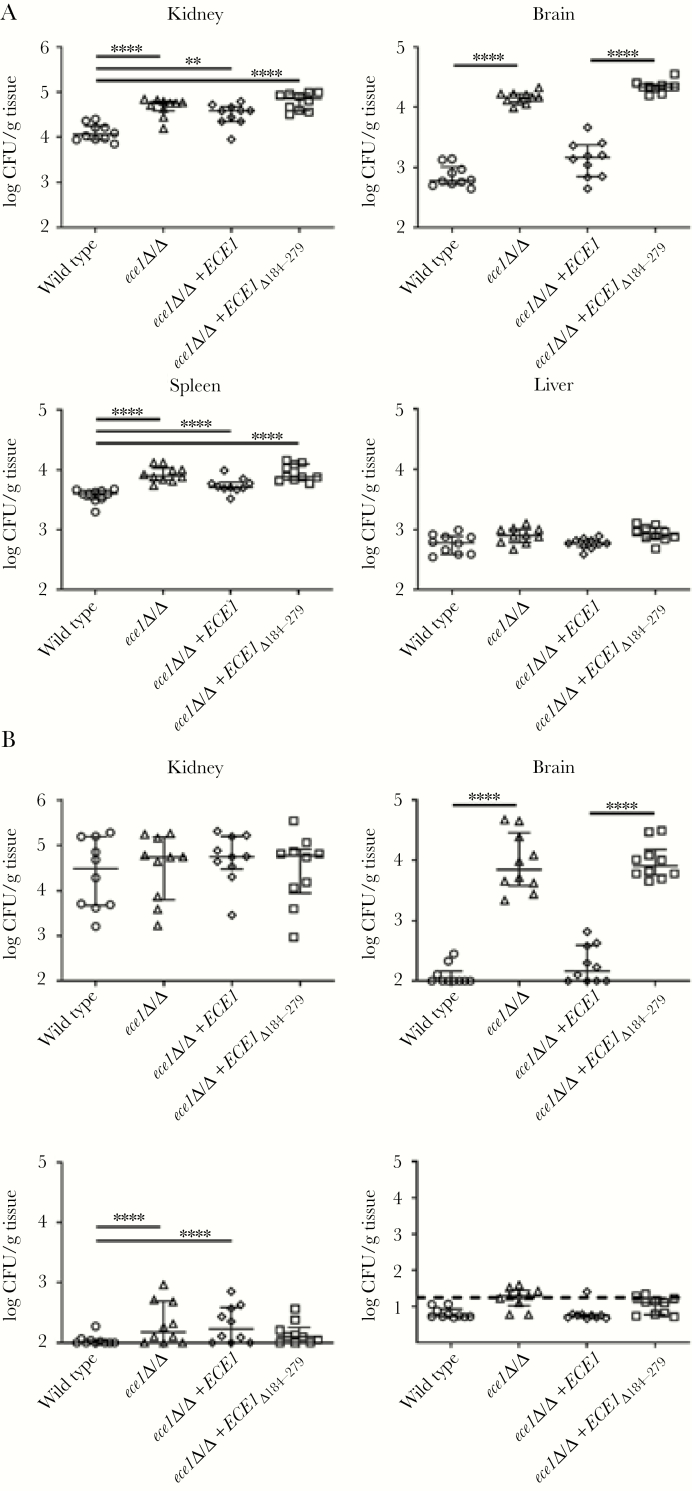
Absence of candidalysin increases organ-specific tropism during hematogenously disseminated candidiasis. Fungal burden of the spleen, brain, kidney, and liver of mice at 1 day (A) and 4 days (B) postinfection after inoculation with 2 × 10^5^*Candida albicans* cells of the indicated strains. Results are median ± interquartile range of 2 independent experiments with 10 mice per strain at each time point. Statistical significance is indicated by **, *P *< .01 and ****, *P *< .0001 (Mann-Whitney test with Bonferroni correction for multiple comparisons). Dashed line indicates limit of detection in the liver. CFU, colony-forming units.

The zebrafish screen demonstrated the importance of candidalysin in promoting *C albicans-*mediated infection. Therefore, we progressed to determine whether candidalysin was also required for systemic infection in a murine model of disseminated candidiasis. For these experiments, we included an additional *C albicans* strain deficient solely in the candidalysin-encoding region of *ECE1* (*ece1*Δ/Δ+*ECE1*_Δ184–279_). Animals were infected with *C albicans* strains, and fungal burden, tissue invasion, cytokine production, and survival were monitored over time. Fungal burdens were monitored at day 1 and 4 postinfection in the spleen, brain, kidney, and liver (Figure 3). Also of note, at day 1, there was a significant increase in burdens in the spleen, brain, and kidney of animals infected with both candidalysin-deficient *C albicans* strains (*ece1*Δ/Δ, *ece1*Δ/Δ+*ECE1*_Δ184–279_) in comparison with the wild-type strain (BWP17+CIp30). A significant increase in burdens was also observed in the spleen and kidney of animals infected with the *ECE1* revertant strain (*ece1*Δ/Δ*+ECE1*). By day 4, there were no differences in burdens between the *C albicans* strains, except for in the brain, where burdens remained high in animals infected with candidalysin-deficient *C albicans* strains (2 logs greater than wild-type). There was no difference in burdens between *C albicans* strains on day 1 or 4 in the liver.

**Figure 4.**
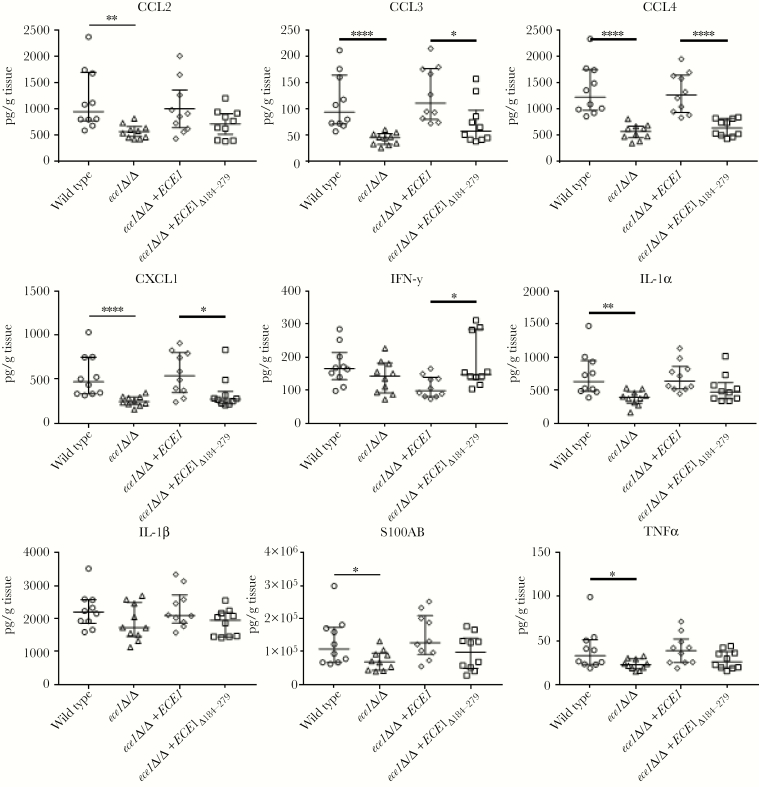
Candidalysin is required to induce an early antifungal immune response in the kidney. Level of chemokines and cytokines in kidney homogenates of immunocompetent mice with hematogenously disseminated candidiasis after 1 day of infection with 2 × 10^5^*Candida albicans* cells of the indicated strains. Results are median ± interquartile range of 2 independent experiments with 10 mice per strain. Statistical significance is indicated by *, *P *< .05, **, *P *< .01, and ****, *P *< .0001 (Mann-Whitney test with Bonferroni correction for multiple comparisons).

Given that the main differences in fungal burdens between the *C albicans* strains was at day 1, we assessed the proinflammatory response in kidney homogenates at this time point (Figure 4). In comparison to wild-type *C albicans*, the *ECE1*-deficient strain (*ece1*Δ/Δ) induced significantly decreased levels of CCL2, CCL3, CCL4, CXCL1, IL-1α, S100A8, and TNFα, which was mirrored by the candidalysin-deficient strain (*ece1*Δ/Δ+*ECE1*_Δ184–279_) for CCL3, CCL4, and CXCL1. However, although not always statistically significant, there was a clear trend of reduced secretion of all cytokines, except interferon-γ, with *ece1*Δ/Δ+*ECE1*_Δ184–279_. Also of note, all cytokine responses recovered to wild-type levels with the *ece1*Δ/Δ+*ECE*1 revertant strain. The data indicate that candidalysin is a key driver of proinflammatory responses during systemic infection.

**Figure 5.**
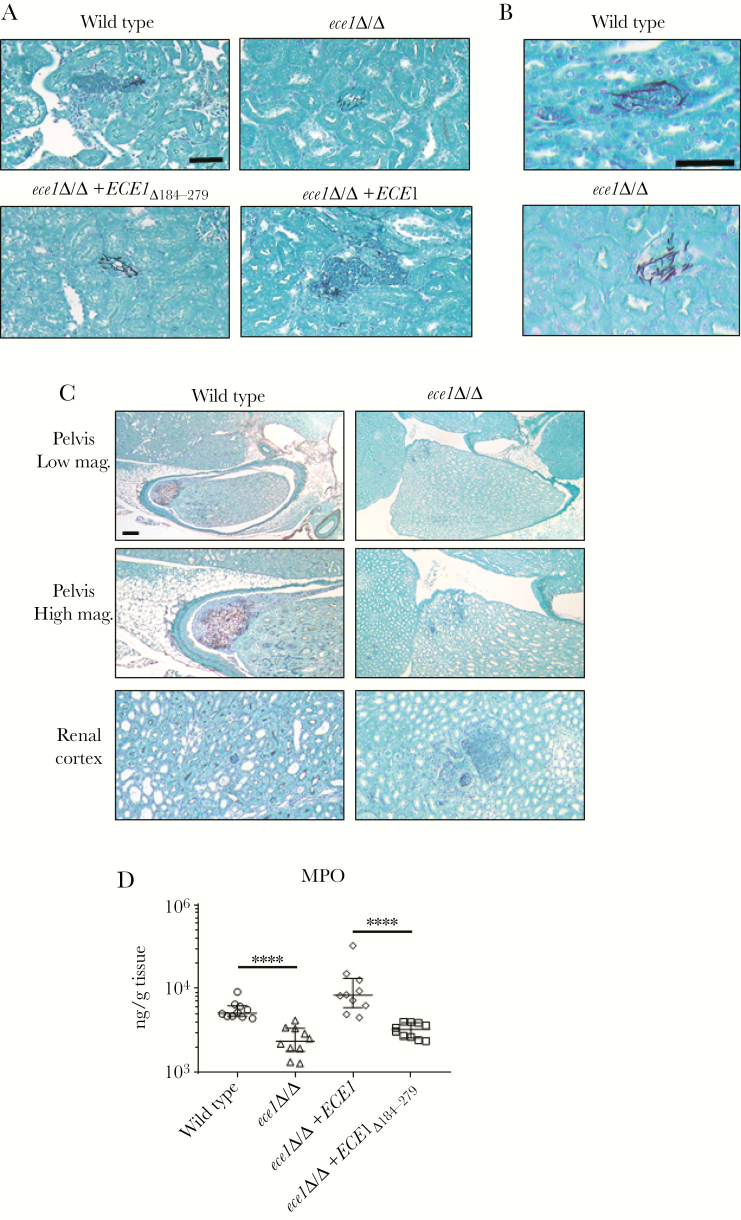
Candidalysin is required for renal neutrophil recruitment and immunopathology. (A and B) Histopathology of the kidney of mice after 1 day of infection with 2 × 10^5^*Candida albicans* cells of the indicated strains. Sections (A, low magnification; B, high magnification) were stained with Gomori methenamine silver (GMS). Fungal cells are filamentous and stained black, and polymorphonuclear (PMN) cells are round and dark purple. Scale bar = 50 µm. (C) Histopathology of the pelvis region (top and middle) and renal cortex (bottom) of the mouse kidney 4 days postinfection with 2 × 10^5^*C albicans* cells of the indicated strains. Sections were stained with GMS. Fungal cells are filamentous and stained black, and PMN cells are round and purple surrounding fungal filaments. Only wild-type *C albicans* invades the pelvis region with local immunopathology, whereas the *ece1*Δ/Δ remains in the renal cortex. Scale bar = 200 µm. (D) Myeloperoxidase (MPO) expression in kidney homogenates after 1 day of infection with 2 × 10^5^*C albicans* cells of the indicated strains. Results are median ± interquartile range of 2 independent experiments with 10 mice per strain. Statistical significance is indicated by ****, *P *< .0001 (Mann-Whitney test with Bonferroni correction for multiple comparisons).

The key role of candidalysin in damaging and inducing neutrophil-recruiting chemokines (eg, CXCL1, CXCL8) in mice and human endothelial cells prompted us to analyze the invasion and neutrophil recruitment capabilities of the *C albicans* strains by histology. All *C albicans* strains were able to form hyphae and invaded the kidney at day 1 postinfection (Figure 5A and B). However, only the candidalysin-producing strains induced strong neutrophil accumulation to the infection site. At later time points during hematogenously disseminated candidiasis, inflammatory processes spread to the renal tubules and pelvis with marked neutrophil accumulation, which can mediate tissue damage [36, 37]. Therefore, we compared the ability of the wild-type and *ece1*Δ/Δ *C albicans* strains to extend to the pelvis region of the kidney and cause renal pelvis injury at day 4 postinfection (Figure 5C) when similar fungal burdens were observed (Figure 3B). Only wild-type *C albicans* was able to invade the pelvis region, with concomitant neutrophil accumulation and associated local immunopathology, whereas *ece1*Δ/Δ was unable to invade the pelvis region and remained in the renal cortex (Figure 5C). The increased ability of candidalysin-producing *C albicans* strains to recruit neutrophils was confirmed by the increased production of MPO in kidney homogenates (Figure 5D). The data indicate that during systemic infection, candidalysin promotes *C albicans* invasion and is a potent mediator of neutrophil recruitment to the infection site.

**Figure 6.**
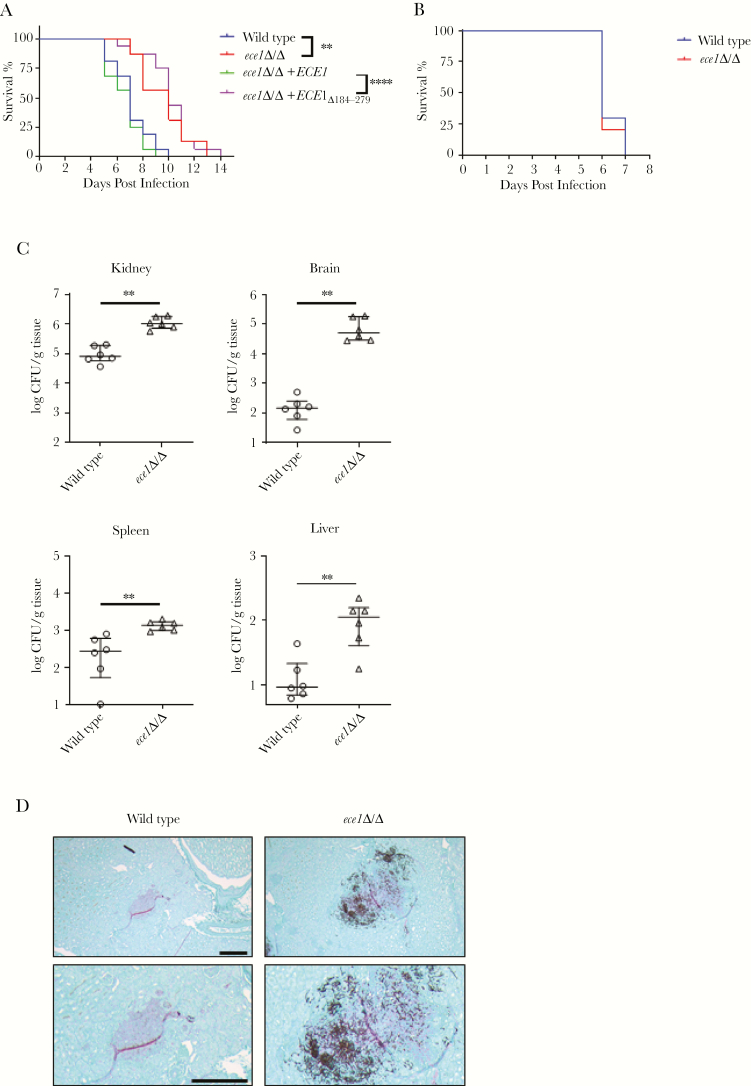
Candidalysin-induced immunopathology is required for virulence during hematogenously disseminated candidiasis. (A) Survival of immunocompetent mice after intravenous inoculation with 2 × 10^5^ yeast phase cells of the indicated strains of *Candida albicans* (n  = 10). (B) Survival of leukopenic mice infected with 5 × 10^4^ wild-type or *ece1*Δ/Δ *C albicans* yeast. Statistical significance is indicated by **, *P *< .01 (wild-type vs *ece1*Δ/Δ) and ****, *P *< .0001 (*ece1*Δ/Δ+*ECE1* vs *ece1*Δ/Δ*+ECE1*_*Δ184–279*_) (Wilcoxon rank-sum test). (C) Fungal burden of the kidney, brain, spleen, and liver of leukopenic mice at 4 days postinfection after inoculation with 5 × 10^4^ wild-type or *ece1*Δ/Δ *C albicans* yeast. Results are median ± interquartile range with 6 mice per strain. Statistical significance is indicated by **, *P *< .01 (Mann-Whitney test). (D) Histopathology of the pelvis region (top, lower magnification; bottom, higher magnification) of the kidney of leukopenic mice after 4 days of infection with 5 × 10^4^*C albicans* cells of the indicated strains. Sections were stained with Gomori methenamine silver. Scale bars = 200 µm. CFU, colony-forming units.

The differences in the capacity of candidalysin-producing and -deficient *C albicans* strains to induce cytokines and neutrophil accumulation suggest that candidalysin plays a key role in driving systemic infection. Therefore, we undertook survival experiments and confirmed that candidalysin-producing strains were more pathogenic than candidalysin-deficient strains. Animals infected with candidalysin-producing strains all succumbed by day 10 (25% survival at day 7), whereas animals infected with candidalysin-deficient strains succumbed by day 14 (100% survival at day 7) (Figure 6A). Given the important function of candidalysin in mediating neutrophil recruitment, we next depleted animals of neutrophils using 5-fluorouracil and assessed the virulence capacity of the wild-type and *ece1*Δ/Δ *C albicans* strains. Under these conditions, both *C albicans* strains became equally virulent and all mice succumbed to infection by day 7 (Figure 6B), despite fungal burdens being significantly higher in all organs infected with *C albicans ece1*Δ/Δ (Figure 6C). As previously shown, *C albicans ece1*Δ/Δ was unable to invade the pelvis region and remained in the renal cortex (Figure 6D). These data, taken together, suggest that candidalysin production during systemic infections induces local proinflammatory and chemotactic responses, which function to recruit neutrophils to the infection site, thereby promoting pathogenicity and disease.

## DISCUSSION

The discovery of candidalysin, the first peptide toxin identified in any human fungal pathogen, has provided a significant advance in our understanding of *C albicans* pathogenesis [15, 18, 27–30]. Candidalysin is produced by hyphae and is critically important for promoting mucosal *C albicans* infections [15–17, 19, 24, 25]. Although candidalysin activates the inflammasome in macrophages [13], its role in promoting systemic *C albicans* infection was hitherto unknown. In this study, we demonstrate that candidalysin promotes virulence during systemic *C albicans* infections with a key role in mediating neutrophil accumulation in infected renal tissue.

We first investigated the ability of candidalysin to damage and activate endothelial cells, which may facilitate *C albicans* translocation across the blood vessel lining to establish a hematogenous disseminated infection. Candidalysin damaged endothelial cells and induced MAPK signaling via MEK1/2 and ERK1/2, resulting in the activation of the AP-1 transcription factors, c-Fos and c-jun, and the secretion of the neutrophil-recruiting chemokine CXCL8. Although candidalysin activates similar MAPK/c-Fos-mediated pathways in oral [15, 19, 24] and vaginal [16] epithelial cells, the induction of the MAPK/c-jun pathway appears specific to endothelial cells. Given that CXCL8 expression is mediated by c-jun/AP-1 in endothelial cells [38], the data indicate that candidalysin activates c-jun/AP-1 to upregulate CXCL8 secretion. Therefore, although both endothelial and epithelial cells respond to candidalysin in a similar fashion, some cell-specific differences are observable. However, we note that these experiments were undertaken in vitro and acknowledge that evidence for a direct effect of candidalysin on endothelial cell activation events in vivo is limited from these studies.

In previous studies, 2 other fungal proteins, Ssa1p and Als3p, were shown to mediate *C albicans* endocytosis in endothelial cells via N-cadherin [6, 7, 39]. Although immune activation mechanisms by Ssa1 has not been investigated, Als3p does not induce MAPK/c-Fos signaling or cytokine release in oral epithelial cells [40] and is dispensable for virulence in murine [41] and zebrafish (this work) models of disseminated candidiasis. Therefore, although *C albicans* possesses multiple hypha-associated factors to promote virulence, candidalysin appears to be the critical factor inducing endothelial cell damage and proinflammatory cytokines that recruit and activate phagocytes, such as neutrophils.

In both zebrafish and murine infection models, candidalysin-producing strains were significantly more lethal than candidalysin-deficient *C albicans* strains. The increase in mortality directly correlated with immune activation, because only candidalysin-producing strains induced strong cytokine/chemokine secretion, particularly those associated with immune cell (notably neutrophil) recruitment (CCL2/3/4, CXCL1, S100A8). Histological and ELISA (MPO) analyses confirmed that only candidalysin-producing strains were able to induce prominent neutrophil accumulation to the kidney at day 1 postinfection, at a time point when neutrophils are protective [42, 43]. In agreement with the early protective effect of neutrophils, mice infected with wild-type *C albicans* had reduced fungal burdens at this time point. In contrast, neutrophils exert detrimental effects in this model at later stages of infection, and part of this immunopathogenic response is driven by the CCL3-CCR1 chemokine axis [37, 44]. Also of note, only wild-type *C albicans* invaded the pelvic region of the kidney at day 4, which correlated with significant neutrophil accumulation and associated tissue injury, despite the presence of similar fungal burdens in the mouse kidneys infected with the *ECE1*-deficient *C albicans* strain. Instead, infection with candidalysin-deficient strains resulted in decreased CCL3 levels, the ligand for CCR1, and decreased renal pelvis damage and neutrophil-mediated immunopathology. Given that candidalysin is known to promote *C albicans* translocation across the gut barrier [17], the data support the notion that candidalysin may also facilitate *C albicans* invasion through parenchymal organs during systemic infections. Our data correlate (1) with previous data in the systemic candidiasis model indicating that CCR1-mediated neutrophil recruitment at later infection time points can be detrimental [37, 44] and (2) with mucosal infection studies demonstrating that neutrophil recruitment to the site of infection is candidalysin-dependent [15, 16, 24].

The dual role of candidalysin in promoting infection while concurrently inducing neutrophil responses was confirmed in neutrophil-depletion experiments, where both candidalysin-producing and -deficient *C albicans* strains became equally virulent. It is interesting to note that the significantly higher fungal burdens in all organs of leukopenic animals infected with *C albicans ece1*Δ/Δ suggest that candidalysin activity and immune cell activation, but not fungal burdens, are the critical factors in determining morbidity and mortality during systemic infections. The findings are clinically relevant because neutropenic patients are highly susceptible to disseminated candidiasis [3, 45]. As such, the data inextricably link (1) candidalysin activity with *C albicans* pathogenicity and (2) neutrophil activation with disease outcome.

As mentioned above, it is intriguing that mortality and immune activation did not correlate with increased fungal burdens. This was most notable in the brain, where significantly more fungal burdens were observed with candidalysin-deficient strains. These striking differences in fungal burdens were recently found to be due to candidalysin-dependent induction of IL-1β and CXCL1 secretion from CARD9^+^ microglial cells, which function to recruit CXCR2-expressing neutrophils to the brain to control the infection and hence reduce fungal burdens [46]. This finding, together with the fact that candidalysin-deficient strains produce normal hyphae [15], may challenge the long-held view that *C albicans* pathogenicity in systemic infections directly correlates with increased fungal burdens and hypha formation. Indeed, in certain tissues (ie, brain), the data appear to uncouple pathogenicity from hypha formation and correlate pathogenicity with the ability of *C albicans* to damage and induce strong local immune responses, predominantly through candidalysin activity and neutrophil recruitment.

## CONCLUSIONS

The data, taken together, suggest that during disseminated candidiasis, candidalysin production by *C albicans* hyphae stimulates a strong proinflammatory response with neutrophil recruitment, which reduces fungal burdens during early infection but later hastens mortality likely related to immunopathogenic effects. In contrast, in the absence of candidalysin, there is a reduced inflammatory response with a lack of neutrophil recruitment, which promotes *C albicans* proliferation during early infection but also prolongs survival, enabling the fungus to traffic to and survive in the brain. In summary, the data demonstrate that candidalysin production during disseminated systemic infections promotes virulence, neutrophil recruitment, and disease and may identify candidalysin-associated damage and immune activation pathways as novel targets to combat disseminated fungal infections.

## Notes

***Financial support.*** This work was funded by grants from the Wellcome Trust (214229_Z_18_Z), Biotechnology and Biological Sciences Research Council (BB/N014677/1), National Institutes of Health (NIH) (R37-DE022550), King’s Health Partners Challenge Fund (R170501), and the NIH Research at Guys and St. Thomas’s NHS Foundation Trust and the King’s College London Biomedical Research Centre (IS-BRC-1215–20006; to J. R. N.); National Institutes of Health Grants K99DE026856 (to M. S.) and R01DE022600 and R01AI124566 (to S. G. F.). This work was also funded in part by the Division of Intramural Research of the National Institute of Allergy and Infectious Diseases, NIH (to M. S. L.) and the Deutsche Forschungsgemeinschaft under Germany’s Excellence Strategy (EXC 22167-390884018 and the CRC/TR 124 FungiNet project C1; to B. H.).

***Potential conflicts of interest.*** All authors: No reported conflicts of interest. All authors have submitted the ICMJE Form for Disclosure of Potential Conflicts of Interest.

## References

[1] Brown GD, Denning DW, Gow NA, Levitz SM, Netea MG, White TC Hidden killers: human fungal infections. Sci Trans Med 2012; 4:165rv13. 10.1126/scitranslmed.3004404 23253612

[2] Brown GD, Denning DW, Levitz SM Tackling human fungal infections. Science 2012; 336:647. 22582229 10.1126/science.1222236

[3] Pappas PG, Lionakis MS, Arendrup MC, Ostrosky-Zeichner L, Kullberg BJ Invasive candidiasis. Nat Rev Dis Primers 2018; 4:18026. 29749387 10.1038/nrdp.2018.26

[4] Grubb SE, Murdoch C, Sudbery PE, Saville SP, Lopez-Ribot JL, Thornhill MH *Candida albicans*-endothelial cell interactions: a key step in the pathogenesis of systemic candidiasis. Infect Immun 2008; 76:4370–7. 18573891 10.1128/IAI.00332-08 PMC2546854

[5] Filler SG, Sheppard DC Fungal invasion of normally non-phagocytic host cells. PLoS Pathog 2006; 2:e129. 17196036 10.1371/journal.ppat.0020129 PMC1757199

[6] Phan QT, Myers CL, Fu Y, et al Als3 is a *Candida albicans* invasin that binds to cadherins and induces endocytosis by host cells. PLoS Biol 2007; 5:e64. 17311474 10.1371/journal.pbio.0050064 PMC1802757

[7] Sun JN, Solis NV, Phan QT, et al Host cell invasion and virulence mediated by *Candida albicans* Ssa1. PLoS Pathog 2010; 6:e1001181. 21085601 10.1371/journal.ppat.1001181 PMC2978716

[8] Liu Y, Filler SG *Candida albicans* Als3, a multifunctional adhesin and invasin. Eukaryot Cell 2011; 10:168–73. 21115738 10.1128/EC.00279-10 PMC3067396

[9] Seidl K, Solis NV, Bayer AS, et al Divergent responses of different endothelial cell types to infection with *Candida albicans* and *Staphylococcus aureus*. PLoS One 2012; 7:e39633. 22745797 10.1371/journal.pone.0039633 PMC3382135

[10] Müller V, Viemann D, Schmidt M, et al *Candida albicans* triggers activation of distinct signaling pathways to establish a proinflammatory gene expression program in primary human endothelial cells. J Immunol 2007; 179:8435–45. 18056390 10.4049/jimmunol.179.12.8435

[11] Filler SG, Pfunder AS, Spellberg BJ, Spellberg JP, Edwards JE Jr *Candida albicans* stimulates cytokine production and leukocyte adhesion molecule expression by endothelial cells. Infect Immun 1996; 64:2609–17. 8698486 10.1128/iai.64.7.2609-2617.1996 PMC174117

[12] Barker KS, Park H, Phan QT, et al Transcriptome profile of the vascular endothelial cell response to *Candida albicans*. J Infect Dis 2008; 198:193–202. 18500935 10.1086/589516

[13] Kasper L, König A, Koenig PA, et al The fungal peptide toxin Candidalysin activates the NLRP3 inflammasome and causes cytolysis in mononuclear phagocytes. Nat Commun 2018; 9:4260. 30323213 10.1038/s41467-018-06607-1 PMC6189146

[14] Krysan DJ, Sutterwala FS, Wellington M Catching fire: *Candida albicans*, macrophages, and pyroptosis. PLoS Pathog 2014; 10:e1004139. 24967821 10.1371/journal.ppat.1004139 PMC4072798

[15] Moyes DL, Wilson D, Richardson JP, et al Candidalysin is a fungal peptide toxin critical for mucosal infection. Nature 2016; 532:64–8. 27027296 10.1038/nature17625 PMC4851236

[16] Richardson JP, Willems HME, Moyes DL, et al Candidalysin drives epithelial signaling, neutrophil recruitment, and immunopathology at the vaginal mucosa. Infect Immun 2018; 86:e00645–17. 29109176 10.1128/IAI.00645-17 PMC5778364

[17] Allert S, Forster TM, Svensson CM, et al *Candida albicans*-induced epithelial damage mediates translocation through intestinal barriers. mBio 2018; 9:e00915–18. 29871918 10.1128/mBio.00915-18 PMC5989070

[18] Wilson D, Naglik JR, Hube B The missing link between *Candida albicans* hyphal morphogenesis and host cell damage. PLoS Pathog 2016; 12:e1005867. 27764260 10.1371/journal.ppat.1005867 PMC5072684

[19] Richardson JP, Mogavero S, Moyes DL, et al Processing of *Candida albicans* Ece1p is critical for candidalysin maturation and fungal virulence. mBio 2018; 9:e02178–17. 29362237 10.1128/mBio.02178-17 PMC5784256

[20] Moyes DL, Runglall M, Murciano C, et al A biphasic innate immune MAPK response discriminates between the yeast and hyphal forms of *Candida albicans* in epithelial cells. Cell Host Microbe 2010; 8:225–35. 20833374 10.1016/j.chom.2010.08.002 PMC2991069

[21] Moyes DL, Murciano C, Runglall M, Islam A, Thavaraj S, Naglik JR *Candida albicans* yeast and hyphae are discriminated by MAPK signaling in vaginal epithelial cells. PLoS One 2011; 6:e26580. 22087232 10.1371/journal.pone.0026580 PMC3210759

[22] Moyes DL, Murciano C, Runglall M, Kohli A, Islam A, Naglik JR Activation of MAPK/c-Fos induced responses in oral epithelial cells is specific to *Candida albicans* and *Candida dubliniensis* hyphae. Med Microbiol Immunol 2012; 201:93–101. 21706283 10.1007/s00430-011-0209-y PMC3257392

[23] Murciano C, Moyes DL, Runglall M, et al *Candida albicans* cell wall glycosylation may be indirectly required for activation of epithelial cell proinflammatory responses. Infect Immun 2011; 79:4902–11. 21930756 10.1128/IAI.05591-11 PMC3232641

[24] Verma AH, Richardson JP, Zhou C, et al Oral epithelial cells orchestrate innate type 17 responses to *Candida albicans* through the virulence factor Candidalysin. Sci Immunol 2017; 2:eaam8834. 29101209 10.1126/sciimmunol.aam8834 PMC5881387

[25] Verma AH, Zafar H, Ponde NO, et al IL-36 and IL-1/IL-17 drive immunity to oral candidiasis via parallel mechanisms. J Immunol 2018; 201:627–34. 29891557 10.4049/jimmunol.1800515 PMC6039262

[26] Gladiator A, Wangler N, Trautwein-Weidner K, LeibundGut-Landmann S Cutting edge: IL-17-secreting innate lymphoid cells are essential for host defense against fungal infection. J Immunol 2013; 190:521–5. 23255360 10.4049/jimmunol.1202924

[27] Tang SX, Moyes DL, Richardson JP, Blagojevic M, Naglik JR Epithelial discrimination of commensal and pathogenic *Candida albicans*. Oral Dis 2016; 22(Suppl 1):114–9. 26843519 10.1111/odi.12395

[28] Naglik JR, König A, Hube B, Gaffen SL *Candida albicans*-epithelial interactions and induction of mucosal innate immunity. Curr Opin Microbiol 2017; 40:104–12. 29156234 10.1016/j.mib.2017.10.030 PMC5733685

[29] Richardson JP, Ho J, Naglik JR Candida-epithelial interactions. J Fungi 2018; 4:22. 10.3390/jof4010022 PMC5872325 29419738

[30] Richardson JP, Moyes DL, Ho J, Naglik JR Candida innate immunity at the mucosa. Semin Cell Dev Biol 2019; 89:58–70. 29501618 10.1016/j.semcdb.2018.02.026

[31] Ades EW, Candal FJ, Swerlick RA, et al HMEC-1: establishment of an immortalized human microvascular endothelial cell line. J Invest Dermatol 1992; 99:683–90. 1361507 10.1111/1523-1747.ep12613748

[32] Grubb SE, Murdoch C, Sudbery PE, Saville SP, Lopez-Ribot JL, Thornhill MH Adhesion of *Candida albicans* to endothelial cells under physiological conditions of flow. Infect Immun 2009; 77:3872–8. 19581400 10.1128/IAI.00518-09 PMC2738003

[33] Swidergall M, Solis NV, Lionakis MS, Filler SG EphA2 is an epithelial cell pattern recognition receptor for fungal β-glucans. Nat Microbiol 2018; 3:53–61. 29133884 10.1038/s41564-017-0059-5 PMC5736406

[34] Widziolek M, Prajsnar TK, Tazzyman S, Stafford GP, Potempa J, Murdoch C Zebrafish as a new model to study effects of periodontal pathogens on cardiovascular diseases. Sci Rep 2016; 6:36023. 27777406 10.1038/srep36023 PMC5078774

[35] Sanchez AA, Johnston DA, Myers C, Edwards JE Jr, Mitchell AP, Filler SG Relationship between *Candida albicans* virulence during experimental hematogenously disseminated infection and endothelial cell damage in vitro. Infect Immun 2004; 72:598–601. 14688143 10.1128/IAI.72.1.598-601.2004 PMC344013

[36] Lionakis MS, Lim JK, Lee CC, Murphy PM Organ-specific innate immune responses in a mouse model of invasive candidiasis. J Innate Immun 2011; 3:180–99. 21063074 10.1159/000321157 PMC3072204

[37] Lionakis MS, Fischer BG, Lim JK, et al Chemokine receptor Ccr1 drives neutrophil-mediated kidney immunopathology and mortality in invasive candidiasis. PLoS Pathog 2012; 8:e1002865. 22916017 10.1371/journal.ppat.1002865 PMC3420964

[38] Umebashi K, Tokito A, Yamamoto M, Jougasaki M Interleukin-33 induces interleukin-8 expression via JNK/c-Jun/AP-1 pathway in human umbilical vein endothelial cells. PLoS One 2018; 13:e0191659. 29373608 10.1371/journal.pone.0191659 PMC5786299

[39] Phan QT, Fratti RA, Prasadarao NV, Edwards JE Jr, Filler SG N-cadherin mediates endocytosis of *Candida albicans* by endothelial cells. J Biol Chem 2005; 280:10455–61. 15632157 10.1074/jbc.M412592200

[40] Murciano C, Moyes DL, Runglall M, et al Evaluation of the role of *Candida albicans* agglutinin-like sequence (Als) proteins in human oral epithelial cell interactions. PLoS One 2012; 7:e33362. 22428031 10.1371/journal.pone.0033362 PMC3299778

[41] Cleary IA, Reinhard SM, Miller CL, et al *Candida albicans* adhesin Als3p is dispensable for virulence in the mouse model of disseminated candidiasis. Microbiology 2011; 157:1806–15. 21436220 10.1099/mic.0.046326-0 PMC3167918

[42] Lionakis MS, Levitz SM Host control of fungal infections: lessons from basic studies and human cohorts. Annu Rev Immunol 2018; 36:157–91. 29237128 10.1146/annurev-immunol-042617-053318

[43] Romani L, Mencacci A, Cenci E, Del Sero G, Bistoni F, Puccetti P An immunoregulatory role for neutrophils in CD4+ T helper subset selection in mice with candidiasis. J Immunol 1997; 158:2356–62. 9036985

[44] Lionakis MS, Albert ND, Swamydas M, Lee CR, Loetscher P, Kontoyiannis DP Pharmacological blockade of the chemokine receptor CCR1 protects mice from systemic candidiasis of hematogenous origin. Antimicrob Agents Chemother 2017; 61:e02365–16. 10.1128/AAC.02365-16 PMC5328547 27993850

[45] Swerdloff JN, Filler SG, Edwards JE Jr Severe candidal infections in neutropenic patients. Clin Infect Dis 1993; 17(Suppl 2):S457–67. 8274612 10.1093/clinids/17.supplement_2.s457

[46] Drummond RA, Swamydas M, Oikonomou V, et al CARD9+ microglia promote antifungal immunity via IL-1β- and CXCL1-mediated neutrophil recruitment. Nat Immunol 2019; 20:559–70. 30996332 10.1038/s41590-019-0377-2 PMC6494474

